# Intelligent Fault Identification for Rolling Bearings Fusing Average Refined Composite Multiscale Dispersion Entropy-Assisted Feature Extraction and SVM with Multi-Strategy Enhanced Swarm Optimization

**DOI:** 10.3390/e23050527

**Published:** 2021-04-25

**Authors:** Huibin Shi, Wenlong Fu, Bailin Li, Kaixuan Shao, Duanhao Yang

**Affiliations:** 1 College of Electrical Engineering & New Energy, China Three Gorges University, Yichang 443002, China; shihuibin@ctgu.edu.cn (H.S.); skaixuan@ctgu.edu.cn (K.S.); yangduanhao@ctgu.edu.cn (D.Y.); 2 Hubei Provincial Key Laboratory for Operation and Control of Cascaded Hydropower Station, China Three Gorges University, Yichang 443002, China; 3 Hubei Key Laboratory of Hydroelectric Machinery Design & Maintenance, China Three Gorges University, Yichang 443002, China

**Keywords:** fault identification, variational mode decomposition, average refined composite multiscale dispersion entropy, multistrategy enhanced swarm optimization algorithm, support vector machine

## Abstract

Rolling bearings act as key parts in many items of mechanical equipment and any abnormality will affect the normal operation of the entire apparatus. To diagnose the faults of rolling bearings effectively, a novel fault identification method is proposed by merging variational mode decomposition (VMD), average refined composite multiscale dispersion entropy (ARCMDE) and support vector machine (SVM) optimized by multistrategy enhanced swarm optimization in this paper. Firstly, the vibration signals are decomposed into different series of intrinsic mode functions (IMFs) based on VMD with the center frequency observation method. Subsequently, the proposed ARCMDE, fusing the superiorities of DE and average refined composite multiscale procedure, is employed to enhance the ability of the multiscale fault-feature extraction from the IMFs. Afterwards, grey wolf optimization (GWO), enhanced by multistrategy including levy flight, cosine factor and polynomial mutation strategies (LCPGWO), is proposed to optimize the penalty factor *C* and kernel parameter *g* of SVM. Then, the optimized SVM model is trained to identify the fault type of samples based on features extracted by ARCMDE. Finally, the application experiment and contrastive analysis verify the effectiveness of the proposed VMD-ARCMDE-LCPGWO-SVM method.

## 1. Introduction

The operating conditions of industrial equipment are complicated, and rolling bearings are widely employed in the types of machinery that play important roles in industrial systems, such as coal, petrochemical, electric power and other industries [1,2]. Rolling bearings will inevitably cause damage to different degrees when running for a long time. What is worse, a fault in the rolling bearings may result in mechanical failure, causing economic loss and personal injury, and even inducing catastrophic accidents. However, monitoring the health condition of the rolling bearings through appropriate indicators and providing information can greatly reduce the occurrence of failures, and avoid major accidents [3,4].

Generally, once the rolling bearings fail, it is accompanied by vibration and sound. Therefore, using appropriate technology to process the collected vibration or acoustic signals could well detect potential failures [5,6,7]. Feature extraction is a committed step in identifying rolling bearing faults, but the vibration signals present nonlinear and nonstationary characteristics resulting in a limitation in the ability of feature extraction. Since signals are generally affected by noise information, an excellent signal processing method is necessary to eliminate the negative effects of these interferences [8,9,10]. Therefore, various time-frequency signal analysis approaches have been widely employed to extract the features in the fault identification of rolling bearings, including empirical mode decomposition (EMD) [11,12], local mean decomposition (LMD) [13], ensemble empirical mode decomposition (EEMD) [14] and variational mode decomposition (VMD) [15,16]. Many scholars have conducted much research into these methods: EMD is efficient for dealing with nonstationary signals, decomposing complex signals into a series of intrinsic mode functions (IMFs) adaptively, while endpoint effect defects remain. Compared to EMD, LMD has advantages in reducing iteration times and suppressing endpoint effect, which can adaptively decompose signal into a sum of subcomponents [17]. Nevertheless, LMD is complex in calculation and susceptible to sampling frequency, which affects the decomposition errors. To overcome these limitations, EEMD adds a set of white noise to help analyze the original signal [18]. However, the result impairs the purity of the original signal in the feature extraction process. In contrast to the above methods, VMD has excellent performance in signal processing, which avoids the mode mixing problem in EMD, the influence of sampling frequency in LMD and noise effect in EEMD. Furthermore, the capability and advancement of VMD has already been confirmed by preceding studies in engineering applications [19]. Thus, VMD was employed to decompose the nonstationary fault signals here, which laid the foundation for fault pattern recognition in rolling bearings.

It is key to extract fault features from the vibration signals in order to realize machinery equipment fault identification [20,21]. On account of nonstationary signals being decomposed by VMD, fault feature extraction would be successful and effective in this study. Entropy is a physical quantity, representing the regularity and complexity of a system which can reflect the nonlinear characteristics of a vibration signal. For example, permutation entropy (PE) [22], sample entropy (SampEn) [23] and fuzzy entropy (FE) [24,25] are all familiar entropies in the feature extraction of rolling bearing fault identification. The PE concept computes simply and quickly, but the disparity between the signal amplitude values is not adequately taken into account [26]. However, dispersion entropy (DE) [27,28] has the advantages of less influence by mutation signals, which can solve the shortages of slowing the calculation in SampEn and FE. Nevertheless, DE does not take into account sufficiently the relationship information between neighboring amplitudes. Meanwhile, DE is adept in analyzing time series at a single scale, but it may ignore the hidden valuable fault information at other scales. To overcome the drawbacks, previous researchers have made improvements, for example, GRCMMFDE was proposed by Zheng et al. [20] to extract fault features. In this paper, a modified DE, namely average refined composite multiscale dispersion entropy (ARCMDE) is put forward, which can not only preserve the original data effectively, but also enhance the ability of multiscale fault feature extraction of the IMFs by fusing average refined composite multiscale procedures.

It is virtually a pattern recognition issue for identifying rolling bearing faults. Therefore, many pattern recognition methods have been employed in various engineering application problems. For instance, artificial neural network (ANN) [29,30], Bayesian decision [31] and support vector machine (SVM) [32] have been employed in identification issues. Among the above methods, ANN has a strong capacity to deal with pattern recognition problems, while it requires abundant samples and is time-consuming to adjust the network structure parameters. Bayesian decision performs with notable capacity by considering prior probability, yet good accuracy is premised on a prior model with appropriate assumptions. Compared with above methods, SVM requires a small number of samples for training, and has good generalization ability. What is more, it has particular advantages in dealing with nonlinear and multidimensional pattern recognition problems [33]. It can satisfy the classification performance by means of finding an optimal hyperplane. Meanwhile, SVM has been applied for pattern recognition combining with feature extraction in rolling bearing fault identification. Therefore, SVM is explored to implement fault identification here.

The SVM model is easily affected by penalty factor *C* and kernel parameter *g* when performing pattern recognition. To address this issue, many optimization algorithms have been used to optimize the SVM model, for instance, Harris hawks optimization (HHO) [34,35], whale optimization algorithm (WOA) [36], particle swarm optimization (PSO) [37], moth−flame optimization (MFO) [38], differential evolution (DE) [39], sine cosine algorithm (SCA) [40] and grey wolf optimization (GWO) [41]. Although these intelligent optimization algorithms have achieved some favorable results, there are still problems of premature convergence of different degrees. In order to improve convergence precision, an enhanced GWO algorithm (LCPGWO) coupled with levy flight [42], cosine factor and polynomial mutation [43] is proposed in this paper. Compared with PSO, GWO, SCA, WOA, MFO and DE algorithms on 12 well-known benchmark functions, the results show that the LCPGWO has greater advantages in finding the optimal solution, which is employed to optimize the penalty factor *C* and kernel parameter *g* of SVM in this study.

In conclusion, firstly, the nonstationary original vibration signals were decomposed into several IMFs by means of VMD. Afterwards, ARCMDE was proposed to construct the feature vectors of different fault samples. Subsequently, the LCPGWO was explored to optimize the SVM model, which was employed to carry out the classification of different fault samples. Lastly, VMD-ARCMDE-LCPGWO-SVM method was applied to compare with other methods in terms of different locations and the motor speeds of rolling bearing faults. The performance of the proposed method was proved to be perfect for the engineering application problem. This study has the following contributions:

(1) Average refined composite multiscale dispersion entropy (ARCMDE) was proposed to enhance the ability of fault feature extraction.
(2) A novel multistrategy enhanced swarm optimizer (LCPGWO) was proposed to calibrate the parameters of SVM, which made it an excellent fault identification model.
(3) The effectiveness of LCPGWO was verified by performance analysis with 12 well-known benchmark functions.
(4) The superiority of the proposed fault identification method was ascertained by engineering experiment and comparative analysis.

The rest of this paper is arranged like this. Section 2 contributes to the fundamental theories about VMD and SVM. The proposed fault identification method according to ARCMDE and LCPGWO optimization approach is presented in Section 3. Section 4 is devoted to demonstrate the superiority of the proposed method in engineering application. The conclusions are in Section 5.

## 2. Fundamental Theories

### 2.1. Variational Mode Decomposition

VMD is a nonrecursive signal preprocessing, which can adaptively decompose the nonstationary signal into *K* band-limited intrinsic mode functions (IMFs) by setting the mode number *K* previously. The core of the VMD method is employed to construct and solve variational problems, which is established below:

(1) $$\begin{array}{l} \underset{m_{k},\omega_{k}}{\min}\{\sum_{k}{\left\|\partial_{t}\left[\left(\delta (t)+\frac{j}{\pi t}\right)*m_{k}(t)\right]e^{-j\omega_{k}t}\right\|}_{2}^{2}\}\\ s.t.\;\;\;\sum_{k=1}^{K}m_{k}(t)=f(t),\;\;\;\;\;\;k=1,2,\ldots ,K \end{array}$$

where $m_{k}=\left\{m_{1},m_{2},\ldots ,m_{k}\right\}$ and $\omega_{k}=\left\{\omega_{1},\omega_{2},\ldots ,\omega_{k}\right\}$ represent the set of *K* mode functions and central frequencies, respectively. The $\partial_{t}$ is the partial derivative of time t, δ(t) is the unit pulse function and f(t) is the input signal of the given real value.

The above variational problem can transform into an unconstrained problem, which can be expressed as:

(2) $$\begin{array}{l} L(m_{k},\omega_{k},\beta )=\alpha \sum_{k}{\left\|\partial_{t}\left[\left(\delta (t)+\frac{j}{\pi t}\right)*m_{k}(t)\right]e^{-j\omega_{k}t}\right\|}_{2}^{2}+\;\\ \;\;\;\;\;\;\;\;\;\;\;\;\;\;\;\;{\left\|f(t)-\sum_{k}m_{k}(t)\right\|}_{2}^{2}+\langle \beta (t),f(t)-\sum_{k}m_{k}(t)\rangle \end{array}$$

where α represents the penalty factor and β(t) is the Lagrange multiplier [44].

Then $m_{k}$ and $\omega_{k}$ can be optimized by Equations (3) and (4), respectively.

(3) $$m_{k}^{n+1}=\min\left\{\alpha {\left\|\partial_{t}\left[\left(\delta (t)+\frac{j}{\pi t}\right)*m_{k}(t)\right]e^{-j\omega_{k}t}\right\|}_{2}^{2}+{\left\|f(t)-\sum_{i}m_{i}(t)+\frac{\beta (t)}{2}\right\|}_{2}^{2}\right\}$$

(4) $$\omega_{k}^{n+1}=\min\left\{{\left\|\partial_{t}\left[\left(\delta (t)+\frac{j}{\pi t}\right)*m_{k}(t)\right]e^{-j\omega_{k}t}\right\|}_{2}^{2}\right\}$$

The iterative equations in frequency domain are derived as follows:

(5) $${\hat{m}}_{k}^{n+1}(\omega )=\frac{\hat{f}(\omega )-\sum_{i\neq k}{\hat{m}}_{i}(\omega )+\frac{\hat{\beta }(\omega )}{2}}{1+2\alpha {\left(\omega -\omega_{k}\right)}^{2}}$$

(6) $$\omega_{k}^{n+1}=\frac{\int_{0}^{\infty }\omega {\left|{\hat{m}}_{k}(\omega )\right|}^{2}d\omega }{\int_{0}^{\infty }{\left|{\hat{m}}_{k}(\omega )\right|}^{2}d\omega }$$

The Lagrange multipliers are expressed in Equation (7).

(7) $${\hat{\beta }}^{n+1}(\omega )={\hat{\beta }}^{n}(\omega )+\gamma_{1}(f(\omega )-\sum_{k}{\hat{m}}_{k}^{n+1}(\omega ))$$

where $\gamma_{1}$ represents an updating parameter.

The processes of VMD are the following:

Step 1: Initialize $m_{k}{}^{1}$, $\omega_{k}{}^{1}$, $\beta^{1}$, *n* = 1;

Step 2: Start loop, *n* = *n* + 1;

Step 3: Update $m_{k}$ and $\omega_{k}$ on the basis of Equations (5) and (6);

Step 4: Update β according to Equation (7);

Step 5: If $\sum_{k}{\left\|{\hat{m}}_{k}^{n+1}-{\hat{m}}_{k}^{n}\right\|}_{2}^{2}/{\left\|{\hat{m}}_{k}^{n}\right\|}_{2}^{2}<\varepsilon$, stop the loop, else turn to Step 2 for next iteration.

### 2.2. Support Vector Machine

SVM is designed for the two-classification issue, which can solve learning problems with limited samples. For a given sample set $\left\{\left(x_{i},y_{i}\right)|i=1,\;2,\ldots ,n\right\}$, it maps the sample space to higher dimensions, and then a hyperplane is constructed, which can transform the nonlinear problem of the sample space into the linear problem of the feature space to solve. The hyperplane function is defined as follow:

(8) ϖ·x+b=0

where ϖ and b are weight vector and bias parameter, respectively. Equation ϖ·x is the inner product.

For example, in order to correctly identify samples of a binary classification issue, all samples demand the following conditions:

(9) $$\varpi \cdot x_{i}+b\left\{ \begin{array}{l} >1\;for\;y_{i}=1\\ <-1\;for\;y_{i}=-1 \end{array}\right.$$

The maximizing classification interval is $2/{\left\|\varpi \right\|}^{2}$, which is obtained by minimizing ${\left\|\varpi \right\|}^{2}$. Then the slack term ξ and penalty factor *C* are brought into Equation (9) to solve the linear indivisibility problem of SVM model.

(10) $$\left\{ \begin{array}{l} \min f=\frac{1}{2}{\left\|w\right\|}^{2}+C\sum_{i=1}^{n}\xi_{i}\\ s.t.\;y_{i}({\vec{\varpi }}^{T}\cdot x_{i}+b)\geq 1-\xi_{i},\;\;i=1,2,\ldots ,n \end{array}\right.$$

Lagrange function is introduced in Equation (10), which can be described as:

(11) $$\begin{array}{l} \max L=\sum_{i=1}^{n}\mu_{i}-\frac{1}{2}\sum_{i,j=1}^{n}\mu_{i}\mu_{j}y_{i}y_{j}K(x_{i},x_{j})\\ s.t.\;\sum_{i=1}^{n}\mu_{i}y_{i}=0,\;\;\mu_{i}\geq 0,\;\;i=1,2,\ldots ,n \end{array}$$

where $\mu_{i}$ means the Lagrange multiplier, $K(x_{i},x_{j})$ is the kernel function of SVM.

In this paper, the radial basis function (RBF) is selected as the kernel function of SVM. Solving the dual problem of Equation (11), the optimal classification discriminant function with RBF is defined as:

(12) $$f(x)=sgn(\sum_{i=1}^{n}\mu_{i}K(x_{i},x)+b)$$

Among them, RBF function is represented as:

(13) $$K(x_{i},x_{j})=\phi (x_{i})\cdot \phi (x_{j})=\exp(-g||x_{i}-x_{j}|{|}^{2})$$

where g represents the kernel parameter, ϕ(x) is the nonlinear vector function.

## 3. Intelligent Fault Identification for Rolling Bearings Fusing the Proposed Method

### 3.1. Average Refined Composite Multiscale Dispersion Entropy

#### 3.1.1. Dispersion Entropy

For a given time series $r=\left\{r_{i},i=1,2,\cdots N\right\}$, which length is *N*. $r_{i}$ is normalized by mean of employing a mapping function [27].

(14) $$y_{i}=\frac{1}{\sigma \sqrt{2\pi }}\int_{-\infty }^{r_{i}}e^{\frac{-{\left(s-\mu \right)}^{2}}{2\sigma^{2}}}ds$$

where σ represents the variance of the normal distribution and μ represents the expectation value. The time series r is normalized to $y=\left\{y_{1},y_{2},\cdots y_{n}\right\},y_{i}\in (0,1)$. Subsequently, the phase space is reconstructed into a matrix for **y**:

(15) $$y_{j}^{m}=[y_{j},y_{j+t_{d}},\ldots ,y_{j+(m-1)t_{d}}]$$

where $j=1,2,\cdots ,N-(m-1)t_{d}$, m is embedding dimension, $t_{d}$ is time delay, $y_{j}^{m}$ is mapped to the scope [1,c]:

(16) $$z_{i}^{c}=round(c\cdot y_{i}+0.5)$$

(17) $$z_{j}^{m,c}=[z_{j}^{c},z_{j+t_{d}}^{c},\ldots ,z_{j+(m-1)t_{d}}^{c}]$$

where $z_{j}^{c}$ represents the *j*-th member of class sequence $z_{j}^{m,c}$, and round is rounding. Each $z_{j}^{m,c}$ corresponds to a dispersion pattern $\pi_{v_{0}v_{1}\ldots v_{m-1}}$ with $z_{j}^{c}=v_{0},\;z_{j+d}^{c}=v_{1},\ldots ,\;z_{j+(m-1)\tau }^{c}=v_{m-1}$.

The frequency of $\pi_{v_{0}v_{1}\ldots v_{m-1}}$ can be deduced as:

(18) $$p=\frac{Number\{j|j\leq N-(m-1)t_{d},\pi_{v_{0}v_{1}\ldots v_{m-1}}\}}{N-(m-1)t_{d}}$$

where $Number\{j|j\leq n-(m-1)t_{d},\pi_{v_{0}v_{1}\ldots v_{m-1}}\}$ is the emergence number of each $\pi_{v_{0}v_{1}\ldots v_{m-1}}$ that corresponding to $z_{j}^{m,c}$:

The dispersion entropy is defined as:

(19) $$DE(r,m,c,t_{d})=-\sum_{\pi =1}^{c^{m}}p\cdot \ln(p)$$

There is a linear negative correlation between DE value and time series. The larger the DE value, the more irregular the time series.

#### 3.1.2. Average Refined Composite Multiscale Dispersion Entropy

As a single scale method, DE may result, in that much useful and significant information hides in multiple scales, which would limit the representation precision of nonstationary fault signals. To solve the disadvantage, average refined composite multiscale dispersion entropy (ARCMDE) is proposed, which is utilized to extract multiscale fault features from IMFs. Given time series $r=\left\{r_{i},i=1,2,\cdots N\right\}$ with length N, the *k*-th composite multiscale coarse-grained sequence $u_{k}^{\left(\tau \right)}=\left\{u_{k,1}^{\left(\tau \right)},x_{k,2}^{\left(\tau \right)},\cdots \right\}$ is defined as:

(20) $$u_{k,j}^{\left(\tau \right)}=\frac{1}{\tau }\sum_{i=k+(j-1)\tau }^{k+j\tau -1}u_{i},1\leq j\leq \left|\frac{N}{\tau }\right|,1\leq k\leq \tau$$

where τ is scale factor.

For each scale factor, refined composite multiscale dispersion entropy (RCMDE) is expressed in Equation (21).

(21) $$\begin{array}{l} RCMDE(u,m,c,t_{d},\tau )=-\sum_{\pi =1}^{c^{m}}\bar{p}(\pi_{v_{0}v_{1}\cdots v_{m-1}})\cdot \ln(\bar{p}(\pi_{v_{0}v_{1}\cdots v_{m-1}}))\\ \bar{p}(\pi_{v_{0}v_{1}\cdots v_{m-1}})=\frac{1}{\tau }\sum_{k-1}^{\tau }p_{k}^{\left(\tau \right)} \end{array}$$

where $\bar{p}(\pi_{v_{0}v_{1}\cdots v_{m-1}})$ is mean probability of the dispersion pattern π of coarse-grained sequence $r_{k}^{\left(\tau \right)}$.

Finally, ARCMDE is expressed as average value of all RCMDE in the scale τ, such that:

(22) $$ARCMDE(r,m,c,t_{d},\tau )=\frac{1}{\tau }\sum_{k=1}^{\tau }RCMDE(u,m,c,t_{d},\tau )$$

### 3.2. GWO Coupled with Multiple Enhancement Strategies

#### 3.2.1. Grey Wolf Optimization

Grey wolves are in a dominant position in the competitive natural environment which have a strict social hierarchy and ingenious cooperative predation. According to the behavior of grey wolves when they hunt, GWO is proposed to solve optimization problems, where the grey wolves are graded into four levels [45]. The first level, called α wolf, may not be the strongest wolf, but it is the best manager in the system and responsible for overall planning. The β wolf on the second level is the best substitute for the α wolf. The δ wolf on the third level acts as the suboptimal solution. The ω wolf is the candidate solution at the bottom and is responsible for balancing the internal relations of the wolf population.

The mathematical expression of grey wolves’ predation can be expressed as follows:

(23) $$D=\left|C\cdot X_{p}(t)-X(t)\right|$$

(24) $$X(t+1)=X_{p}(t)-A\cdot D$$

where *D* represents the distance between the wolf and the prey, X and $X_{p}$ denote the position of the grey wolf and prey, respectively, t is current iterations time.

The coefficient vectors *A* in Equation (23) and *C* in Equation (24) are expressed as follows:

(25) $$A=2a\cdot h_{1}-a$$

(26) $$C=2\cdot h_{2}$$

(27) $$a=2-t*\frac{2}{\max}$$

where *a* is convergence factor, which decreases linearly from 2 to 0, $h_{1}$ and $h_{2}$ are random vectors in [0, 1], max is the maximum number of iterations.

In GWO algorithm, α, β and δ wolves to approach and surround the prey when the prey is identified by the grey wolves. Therefore, the position of the prey can be determined by the position of the grey wolves. The mathematical model for updating the position of each wolf is as follows:

(28) $$\left\{ \begin{array}{l} D_{\alpha }=\left|C_{1}\cdot X_{\alpha }(t)-X(t)\right|\\ D_{\beta }=\left|C_{2}\cdot X_{\beta }(t)-X(t)\right|\\ D_{\delta }=\left|C_{3}\cdot X_{\delta }(t)-X(t)\right| \end{array}\right.$$

(29) $$\left\{ \begin{array}{l} X_{1}=X_{\alpha }-A_{1}\cdot D_{\alpha }\\ X_{2}=X_{\beta }-A_{2}\cdot D_{\beta }\\ X_{3}=X_{\delta }-A_{3}\cdot D_{\delta } \end{array}\right.$$

where $D_{\alpha }$, $D_{\beta }$ and $D_{\delta }$ represent distances of α, β and δ wolves from other individuals respectively, $X_{1}$, $X_{2}$ and $X_{3}$ denote the current position of α, β and δ wolves respectively.

The positional relationship between the grey wolf individual ω and the prey can be determined as follows:

(30) $$X(t+1)=\frac{X_{1}+X_{2}+X_{3}}{3}$$

If |A|<1, the wolves will attack the prey; otherwise, the wolves will search for the prey. To sum up, the pseudocode of GWO algorithm is shown in Algorithm 1.
**Algorithm 1.** The algorithm pseudocode of GWO.Initialize grey wolf population $X_{i}(i=1,2,3\cdots ,n)$Initialize the parameters *a*, *A* and *C*Evaluate the fitness of each wolfAssign the best three grey wolves to $X_{\alpha }$, $X_{\beta }$, $X_{\delta }$**while***t < max* iteration**for** each search agentUpdate the position of current grey wolves by Equation (30)**end for**Update *a*, *A* and *C*Evaluate the fitness of each wolfUpdate $X_{\alpha }$, $X_{\beta }$, $X_{\delta }$*t* = *t* + 1**end while**return $X_{\alpha }$

#### 3.2.2. Grey Wolf Optimization Coupled with Multiple Enhancement Strategies

GWO is slow in convergence speed, resulting in an easy fall into local optimum in the later iteration. In this section, the improved GWO coupled with multiple enhancement strategies (LCPGWO) is explored to solve the shortcomings of GWO, which can improve the ability of global search, accelerate convergence and enhance the capacity for preventing local optimum during the later iteration. The realization processes of LCPGWO are described below in detail.

As shown in Equation (25), parameter *a* influences the change of coefficient vectors *A*, which coordinates the local and global explorations. The larger *a* is, the stronger the global exploration ability; the smaller *a* is, the stronger the local exploration ability. To promote the adaptation during both local and global explorations, the linearly decreasing *a* in Equation (27) is substituted with cosine factor as shown in Equation (31). Thus, *a* is large and reduces slowly for global exploration in the early iteration stage, while it will reduce rapidly for the local search in the later iteration stage.

(31) $$a=2\cdot \cos(\frac{\pi }{2}\cdot \frac{t}{\max})$$

Additionally, inertial weight based on cosine factor is introduced in this paper to enhance the global exploration, which can be seen in Equation (32).

(32) $$W=2\cdot \cos(\frac{\pi }{2}\cdot \frac{t}{\max})-1$$

With inertial weight, the positions of α, β and δ wolves are reformulated in Equation (33).

(33) $$\left\{ \begin{array}{l} X_{1}=X_{\alpha }-W\cdot A_{1}\cdot D_{\alpha }\\ X_{2}=X_{\beta }-W\cdot A_{2}\cdot D_{\beta }\\ X_{3}=X_{\delta }-W\cdot A_{3}\cdot D_{\delta } \end{array}\right.$$

In iterative process, it falls into local optimum easily when the ω wolf approaches the other three wolves. By introducing inertial weight [46], the positional relationship between the grey wolf individual ω and the prey can be redefined as follows:

(34) $$X(t+1)=\frac{W_{1}\cdot X_{1}+W_{2}\cdot X_{2}+W_{3}\cdot X_{3}}{3}$$

(35) $$\left\{ \begin{array}{l} W_{1}=\frac{\left|X_{1}\right|}{\left|X_{1}\right|+\left|X_{2}\right|+\left|X_{3}\right|}\\ W_{2}=\frac{\left|X_{2}\right|}{\left|X_{1}\right|+\left|X_{2}\right|+\left|X_{3}\right|}\\ W_{3}=\frac{\left|X_{3}\right|}{\left|X_{1}\right|+\left|X_{2}\right|+\left|X_{3}\right|} \end{array}\right.$$

where $W_{1}$, $W_{2}$ and $W_{3}$ represent the learning rate of ω to α, β and δ wolves, respectively.

In this paper, α wolf is searched globally by levy flight strategy for preventing local optimum, where the flight step is stable expansion distribution. The next generation of α wolf is calculated as follows:

(36) X(t+1)=X(t)+d⊕Levy(θ)

where *X*(*t*) is position of α wolf at *t*-th iteration, operator ⊕ is entry-wise multiplications, *d* and Levy(θ) are random numbers and step of α wolf respectively, which are determined by Equations (37) and (38).

(37) $$d=d_{0}\cdot (X(t)-X_{\alpha }(t))$$

(38) $$Levy(\theta )∼u=t^{-1-\theta }$$

where $d_{0}$ is a constant, θ is levy index, a random number between 0 and 2, whose value is set at 1.5 here, the flight step of α wolf is a power-law formula.

A more detailed description about levy flight can be summarized as Equation (39).

(39) d⊕Levy(θ)∼0.01u|v|1θ(X(t)−Xα(t))

where *s* and *v* are both normal distributions:

(40) $$\left\{ \begin{array}{l} s∼N(0,\sigma_{s}^{2})\\ v∼N(0,\sigma_{v}^{2}) \end{array}\right.$$

(41) $$\sigma_{s}={\left\{\frac{\gamma (1+\theta )\sin(\pi \theta /2)}{\gamma \theta [\left(1+\theta \right)/2]2^{\left(\theta -1\right)/2}}\right\}}^{1/\theta },\sigma_{v}=1$$

where parameter γ is the standard gamma function.

In swarm intelligence optimization algorithm, it traps in local optimum easily. For this purpose, a polynomial mutation operator for GWO is introduced in this section to promote the exploring ability within the whole situation space, thus to avoid from trapping in local optimum as well as maintain the diversity of solution in the later iteration stage. The mathematical formula of the polynomial mutation is written in Equation (42).

(42) $$X(t+1)=X(t)+\xi (u_{k}-l_{k})$$

where *X*(*t*) is the original optimal individual position, *X*(*t* + 1) is the mutated optimal individual position, $u_{k}$ represents the upper limit of the position and $l_{k}$ is the lower limit of the position.

The parameter ξ is calculated as follows:

(43) $$\xi =\left\{ \begin{array}{l} {\left[2u+(1-2s){\left(1-\xi_{1}\right)}^{\eta +1}\right]}^{\frac{1}{\eta +1}}-1,s\leq 0.5\\ 1-{\left[2(1-s)+2(s-0.5){\left(1-\xi_{2}\right)}^{\eta +1}\right]}^{\frac{1}{\eta +1}},s>0.5 \end{array}\right.$$

where parameter *s* is a random number in [0, 1], η is also [0, 1].

The parameters $\xi_{1}$ and $\xi_{2}$ are deduced in Equation (44).

(44) $$\left\{ \begin{array}{l} \xi_{1}=\left(X(t)-l_{k}\right)/\left(u_{k}-l_{k}\right)\\ \xi_{2}=\left(u_{k}-X(t)\right)/\left(u_{k}-l_{k}\right) \end{array}\right.$$

To sum up, the pseudocode of LCPGWO algorithm is displayed in Algorithm 2.
**Algorithm 2.** The algorithm pseudocode of LCPGWO.Initialize grey wolf population $X_{i}(i=1,2,3\cdots ,n)$Initialize the parameters *a* by Equation (31), and initialize *A* and *C*Evaluate the fitness of each wolfAssign the best three grey wolves to $X_{\alpha }$, $X_{\beta }$, $X_{\delta }$**while***t < max* iteration**for** each search agentUpdate the position of current grey wolves by Equation (34)Calculate the new positions of grew wolves employing the levy flight and polynomial mutation by Equations (36) and (42)**end for**Update *a*, *A* and *C*Evaluate the fitness of each wolfUpdate $X_{\alpha }$, $X_{\beta }$, $X_{\delta }$*t* = *t* + 1**end while**return $X_{\alpha }$

#### 3.2.3. Experimental Study and Results Analysis

##### Benchmark Functions

To prove the effectiveness of the proposed LCPGWO algorithm, six well-known nature-inspired optimization algorithms, including PSO, GWO, SCA, WOA, MFO and DE were applied for comparison. Meanwhile, 12 benchmark functions were selected for optimization experiments as listed in Table 1 and divided into two categories, where F1-F7 were unimodal functions and F8-F12 were multimodal functions [47,48,49]. In Table 1, *Fmin* was the minimum value of each benchmark function. The unimodal functions were mainly employed to test the convergence rate of the algorithms, while the multimodal functions were carried out to test the global exploration ability of the algorithms.

##### Comparison and Analysis with Different Algorithms

The experiment was on a personal computer, which was equipped with Windows 10 system and an Intel(R) Core (TM) CPU at 2.89 GHz and 4 GB memory. The simulation software was MATLAB R2016a.

**Figure 1:**
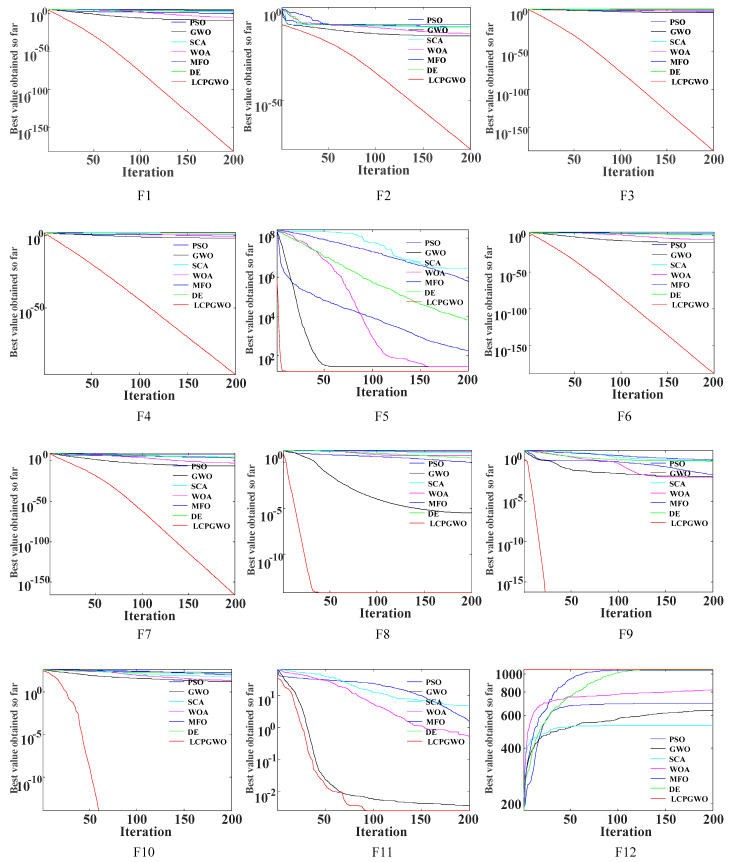
The convergence curves of the seven algorithms on benchmark functions.

Each benchmark function was run 10 times independently to obtain an objective result. The iteration number and searching agents in the experiment were set at 200 and 40, respectively. The detailed parameter settings are shown in Table 2. During iterations of all algorithms, the optimal fitness values were recorded every time. The average fitness values were obtained to draw a curve reflecting the convergence trend of the algorithms. The convergence curves of PSO, GWO, SCA, WOA, MFO, DE and LCPGWO algorithms on the 12 well-known benchmark functions are listed in Figure 1. At the same time, the maximum value, minimum value, mean values and standard deviations of the optimal solution obtained by all algorithms are displayed in Table 3, where a lower value means better search ability and stability.

From Figure 1, it was observed that the proposed LCPGWO algorithm converged better than PSO, GWO, SCA, WOA, MFO, DE algorithms for all F1−F12, indicating that the proposed LCPGWO algorithm was able to prevent local optimum and converge to the optimal value at a faster speed. From Table 3, it can be concluded that the proposed LCPGWO algorithm achieved the lowest value in the maximum value, minimum value, mean value and standard deviation for both unimodal and multimodal functions. In particular, the results of the LCPGWO algorithm were superior to PSO, GWO, SCA, WOA, MFO, DE algorithms, especially on functions F6, F8, F9 and F10. On the whole, the proposed LCPGWO algorithm is more effective and feasible than contrastive methods.

### 3.3. SVM Optimized by LCPGWO

In order to obtain a good generalization performance in dealing with fault identification issues, it is necessary to assign appropriate parameters *C* and *g* of SVM. Thus, the proposed LCPGWO was used to optimize SVM model. The main procedures of classification machine learning with SVM optimized by the proposed LCPGWO algorithm are in below:

Step 1: Initialize the population and set relevant parameters;

Step 2: Update individual’s status according to Equations (36) and (42);

Step 3: Calculate the fitness value, which is the cross-validation accuracy of SVM;

Step 4: Update individual’s new position;

Step 5: Repeat Steps 2–4 until the maximum time of iterations is reached or the convergence condition is met;

Step 6: Choose the maximal cross-validation accuracy as the optimal parameters *C* and *g* of SVM;

Step 7: Train the optimal SVM model according to the training set;

Step 8: Recognize the testing set and finish the identification.

### 3.4. Intelligent Fault Identification for Rolling Bearings Fusing the Proposed Method

**Figure 2:**
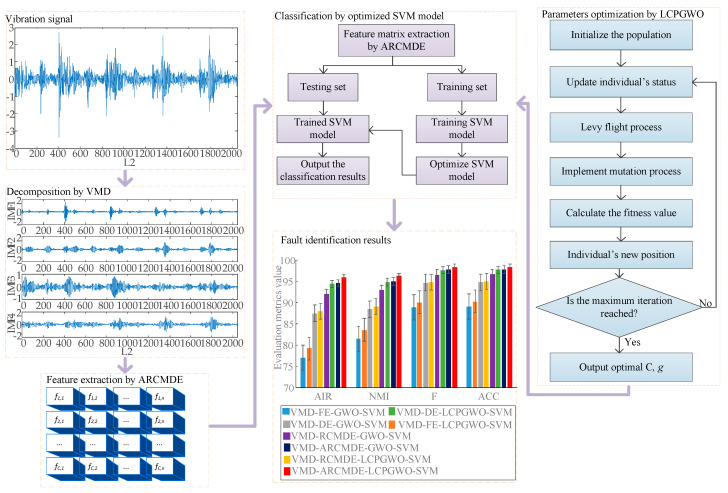
The flowchart of the fault identification with the proposed method.

A novel fault identification method is proposed according to VMD, ARCMDE as the feature extraction and SVM optimized by LCPGWO as classification model in this paper. The flowchart of the fault identification with the proposed method is illustrated in Figure 2. To be specific, firstly, vibration signals were decomposed into four IMFs of different series by VMD. Afterwards, the feature vectors were constructed by means of the proposed ARCMDE, which extracted fault features from IMFs. Finally, the parameters of SVM model were optimized by multistrategy enhanced swarm optimization algorithm LCPGWO, thus achieving the fault pattern recognition.

## 4. Engineering Application

### 4.1. Data Collection

**Figure 3:**
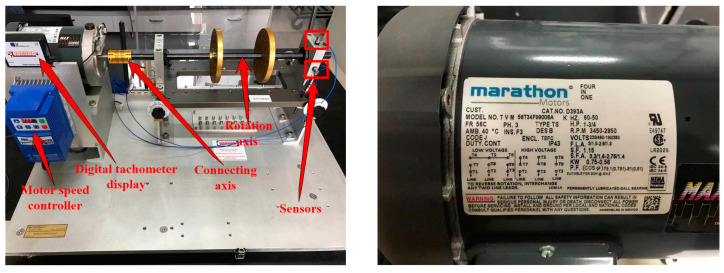
The machinery fault simulator of the rolling bearings.

**Figure 4:**
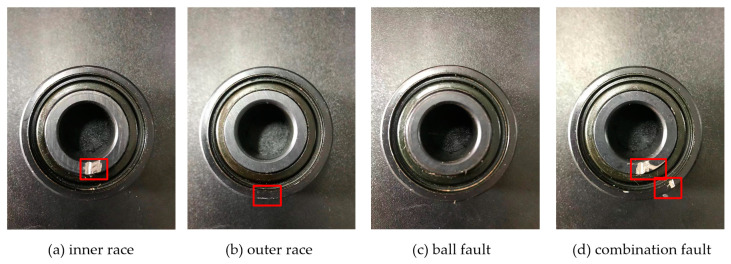
The fault state types of the bearings.

To attest the effectiveness of the proposed method in this paper, the machinery fault simulator (MFS) manufactured by SQI company was used to measure the relevant experimental data for bearings. The detailed information of machinery fault simulator is shown in Figure 3. The type of rolling bearings selected in the experiment was ER12KCL. Meanwhile, the motor speeds of the bearings were 1800 rpm and 2200 rpm when collecting experimental data. The time of sample data collection was set as 10 s. The bearings’ state types were also divided into four, which were inner race fault, ball fault, outer race fault and combination fault, which are displayed in Figure 4. The diameter of all the experimental bearings was 3/4 inches. The vibration signals of the rolling bearings were collected by employing the acceleration sensor mounted on the bearing seat of the motor drive end, and the sampling frequency was 12.8 kHz. Further, there were 61 samples of the vibration signals for each type, and one sample possessed 2048 sample points. The detailed data about the experiments are shown in Table 4.

### 4.2. Application to Fault Identification of Rolling Bearings

To fully prove that the proposed fault identification method was effective, other relevant methods were applied to compare with the proposed VMD-ARCMDE-LCPGWO-SVM method. More specifically speaking, FE, DE and RCMDE were employed to compare with ARCMDE at the feature extraction stage; GWO was applied for comparison at the parameter optimization stage. The settings of the same parameters were uniform in all the comparison experiments.

**Figure 5:**
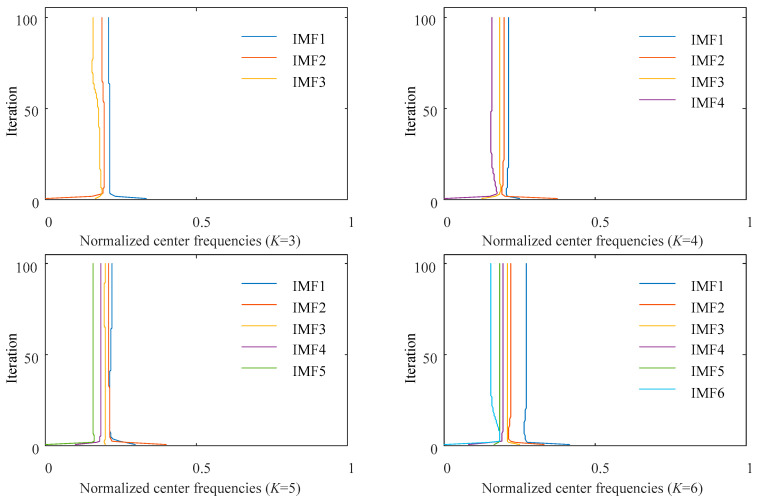
The variation of central frequency with iteration under different *K* values.

Feature extraction is the main problem in the process of identifying rolling bearing faults. VMD was selected to decompose the fault signal into a set of IMFs. The parameter of decomposing mode number *K* was decided in advance, where it was determined by the center frequency observation method according to a previous study [50]. In this paper, the *K* value was obtained by experiment using sample data under a motor speed of 1800 rpm. As shown in Figure 5, if *K* is too large, the center frequencies of adjacent IMFs are too close, resulting in mode mixing, which means excessive decomposition. However, if *K* is too small, the fault signal cannot be effectively decomposed, which leads to more valuable information being ignored. Therefore, the *K* value in the paper was set as 4.

**Figure 6:**
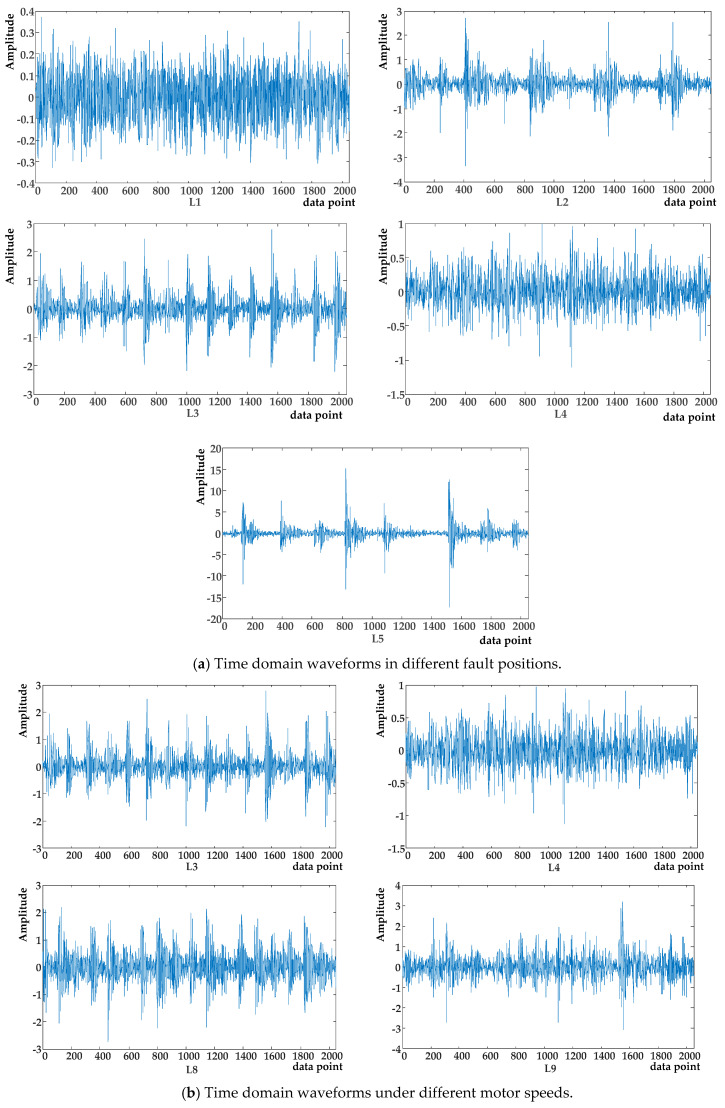

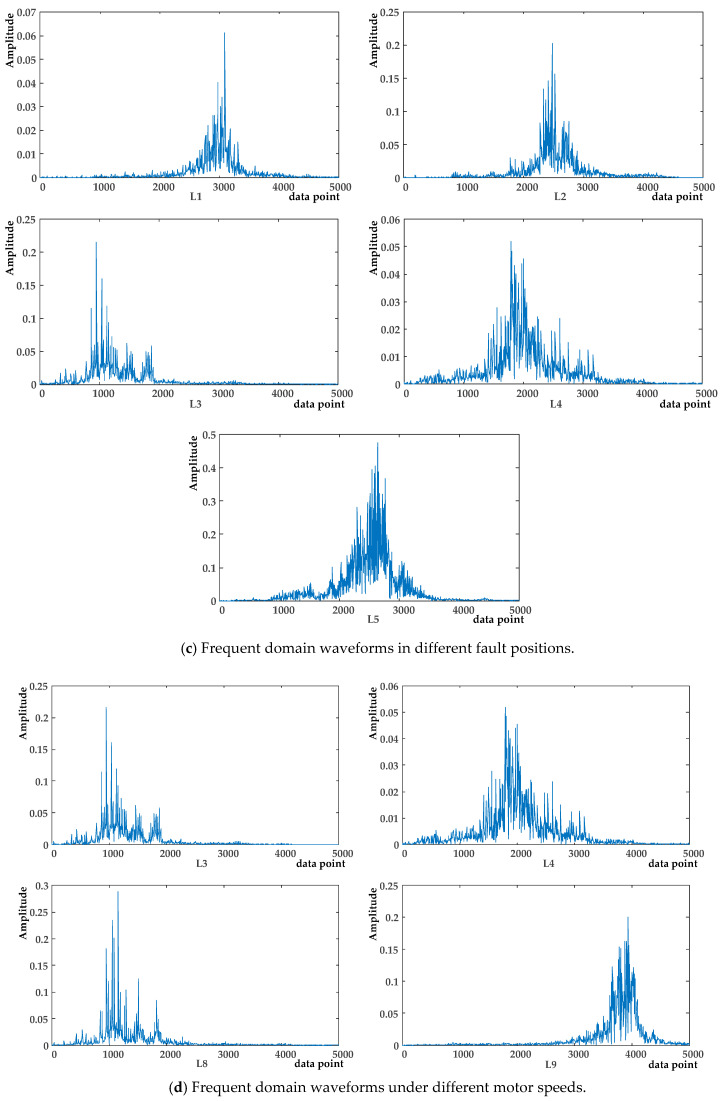
Time and frequent domain waveforms of different signals.

**Figure 7:**
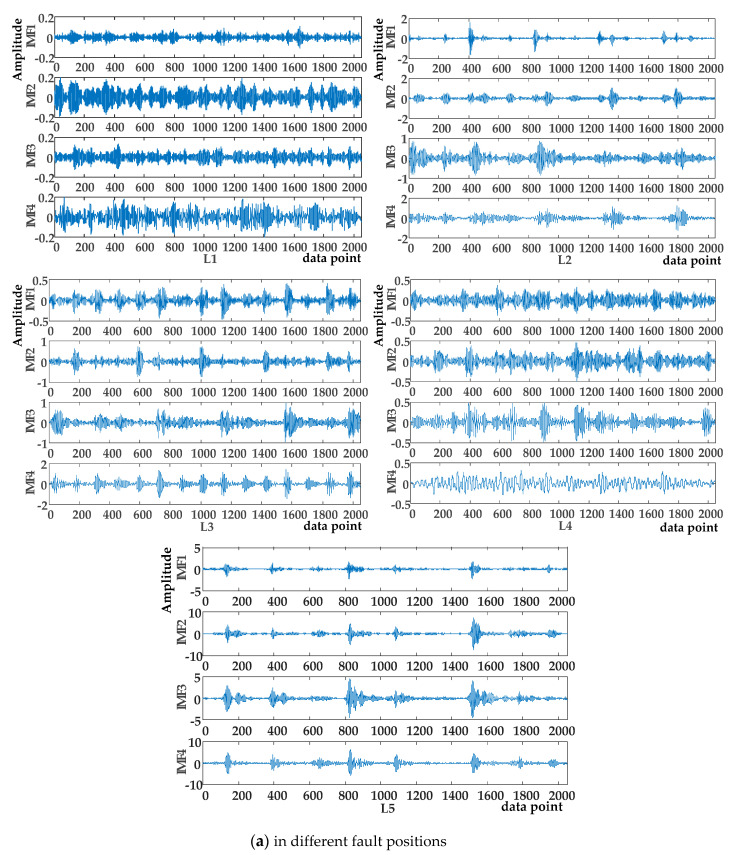

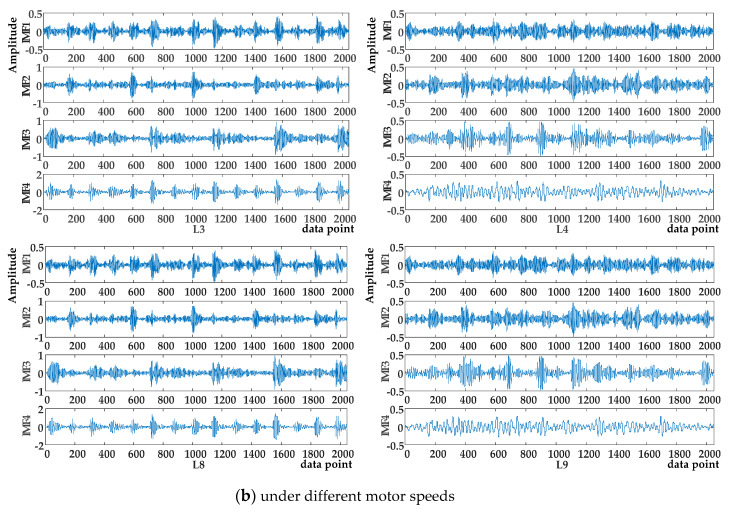
The VMD decomposition results of different signals.

The waveforms of the original signals with different fault positions (L1, L2, L3, L4, L5) and different motor speeds (L3, L4, L8, L9) are illustrated in Figure 6. With VMD decomposition, all the vibration signals were decomposed into four subcomponents including IMF1, IMF2, IMF3, IMF4, as shown in Figure 7. IMFs decomposed from original signals have quite different fluctuation characteristics.

After the IMFs of all samples were obtained through signal decomposition, fault feature vectors were constructed by calculating ARCMDE values, where parameters should be chosen properly beforehand [51,52]. Here, four parameters were set in advance, which were embedding dimension *m*, number of class *c*, maximum scale factor $\tau_{\max}$, and time delay $t_{d}$. By referring to previous papers [53], the parameter settings of ARCMDE are displayed in Table 5.

To verify the performance of the proposed method, 61 feature vectors belonging to different fault types were selected for the contrast experiment. They were randomly divided into two parts, where 40 vectors were selected for training and the remaining 21 vectors were for testing. After that, the proposed LCPGWO method was utilized to enhance the classification identification performance of SVM by searching the optimal values of parameters *C* and *g* of SVM model. The searching range of *C* and *g* were set in [2^−10^, 2^10^], meanwhile, the optimization experiments were accomplished by 100 iterations and 20 searching agents. The five-fold cross-validation was applied for calculating the fitness values of training samples in this experiment. Hence, according to the obtained optimal parameters *C* and *g* by the proposed LCPGWO method, the SVM model was trained and employed to achieve fault identification. For a dependable verification of the proposed method about the effectiveness and superiority, each of these comparative fault identification methods was run 10 times, on average, independently, and training samples were randomly selected. Moreover, accuracy (ACC), adjusted rand index (ARI), F-measure (F) and normalized mutual information (NMI) [54,55] were applied to evaluate the capability of these different approaches. The higher values of the four metrics mean that the matching degree between fault identification result and real samples distribution information is better. The calculation methods of the four metrics are shown in the Table 6, where the range of ARI is set in [−1, 1], and the rest of the metrics are in [0, 1].

The following notations in Table 6 are adopted: TP, TN, FP, FN represent true positive, true negative, false positive, false negative on the basis of the result of fault identification and actual label; Φ and Ω are the sets of given actual label classified result, respectively; *n*_11_ is the number of sample pairs for the same label in both Φ and Ω, while *n*_00_ is for different label, $C_{n}^{2}$ is all possible sample pairs combinations; *P*(𝜑) and *P*(𝜔) represent the probability functions of Φ and Ω respectively; *P*(𝜑,𝜔) is the joint probability functions of Φ with Ω.

Eight relevant methods were employed to compare for illustration of the advantages of the proposed approach in this study. The four evaluation values of fault identification results are shown in Table 7. By comparing with the results of different methods, it proves that the proposed VMD-ARCMDE-LCPGWO-SVM method is the best of four evaluation metrics. It has the highest values at 0.9597, 0.9627, 0.9838, 0.9838 under a motor speed of 1800 rpm and 0.9303, 0.9381, 0.9712, 0.9714 under a motor speed of 2200 rpm. Although the performance of NMI under 2200 rpm was not optimal, it was still very good, which could be considered a desirable result. The evaluation value deviations were also very low. In order to better analyze the results, the results when the motor speed was 1800 rpm were taken as the analysis. For feature extraction, VMD-FE-GWO-SVM, VMD-DE-GWO-SVM, VMD-RCMDE-GWO-SVM and VMD-ARCMDE-GWO-SVM methods were compared, respectively. The ACC of the VMD-ARCMDE-GWO-SVM was 0.9781, which was superior to the VMD-FE-GWO-SVM, VMD-DE-GWO-SVM and VMD-RCMDE-GWO-SVM methods. Similarly, comparing with VMD-FE-LCPGWO-SVM, VMD-DE-LCPGWO-SVM and VMD-RCMDE-LCPGWO-SVM methods, the proposed VMD-ARCMDE-LCPGWO-SVM method also had the highest accuracy. The results revealed that the proposed ARCMDE method was superior for feature extraction.

For the parameter optimization of the SVM model, it can be observed that the ACC of the proposed VMD-ARCMDE-LCPGWO-SVM method was far better than VMD-ARCMDE-GWO-SVM method. Additionally, the VMD-FE-LCPGWO-SVM and VMD-DE-LCPGWO-SVM methods also performed better than the VMD-FE-GWO-SVM and VMD-DE-GWO-SVM methods, respectively, which proved the effectiveness of LCPGWO method for parameter optimization. Based on the above experimental analyses, the conclusion can be drawn that the proposed VMD-ARCMDE-LCPGWO-SVM method achieves stable competitiveness when compared with other methods.

**Figure 8:**
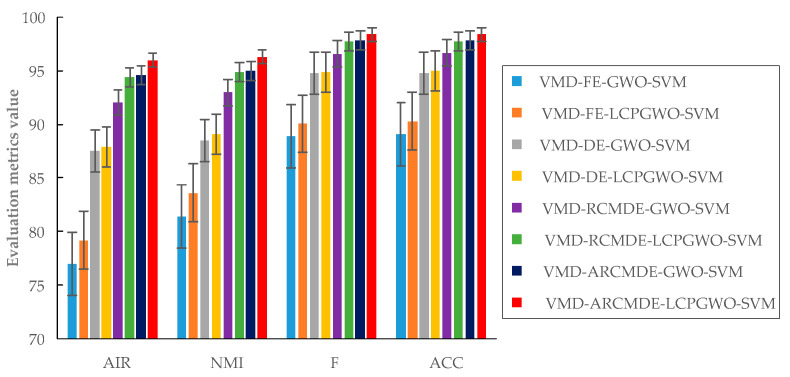
Comparison of evaluation values of different methods under 1800 rpm.

**Figure 9:**
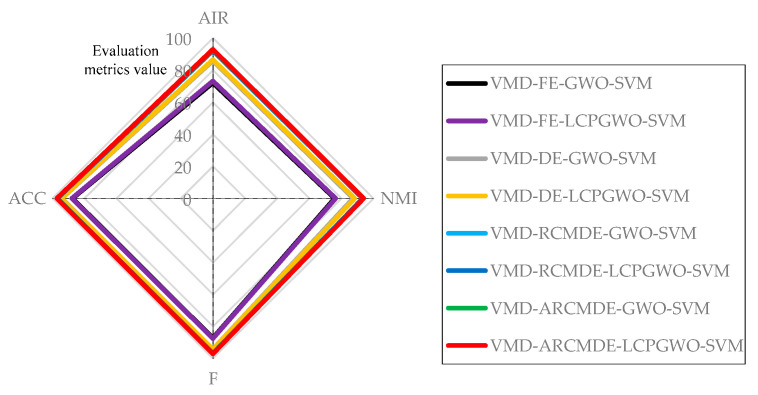
The radar chart of evaluation results with different methods under 2200 rpm.

**Figure 10:**
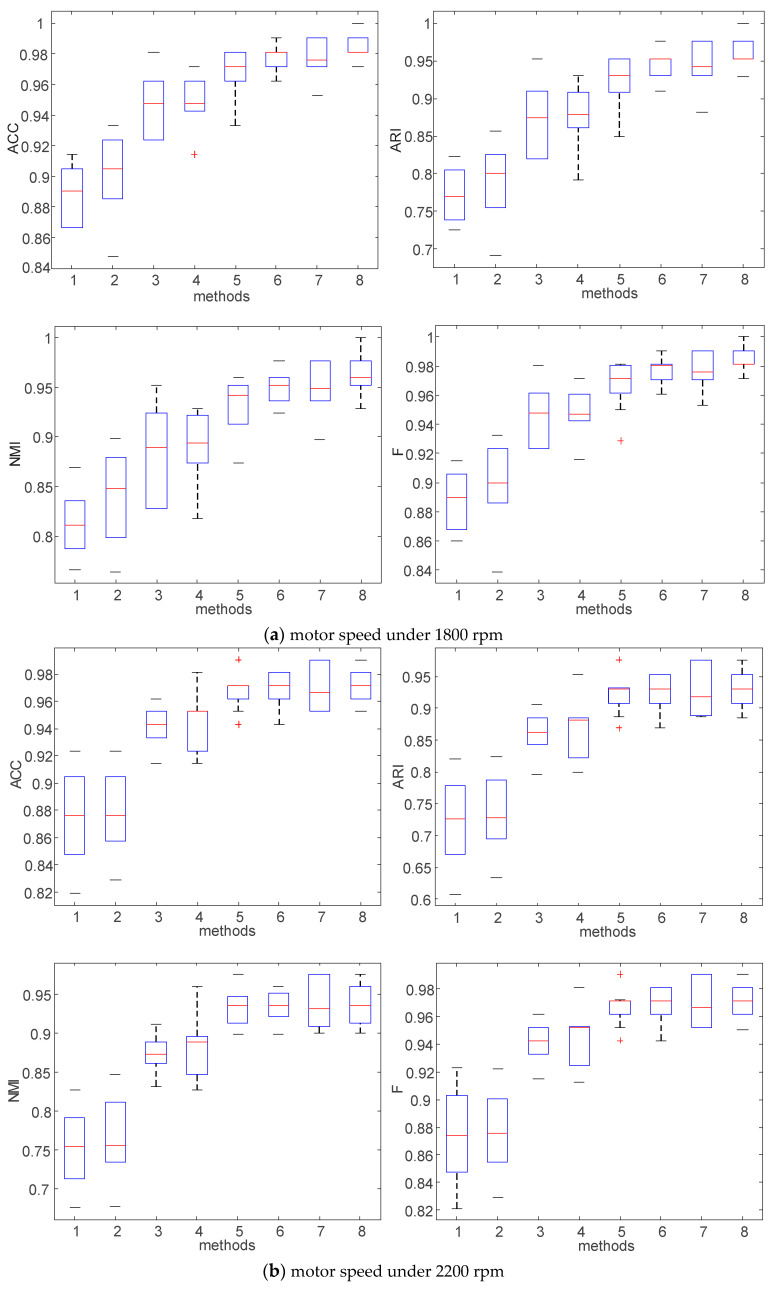
Boxplots of identification results with different methods: the *x*-axis tick labels correspond to: 1: VMD-FE-GWO-SVM; 2: VMD-FE-LCPGWO-SVM; 3: VMD-DE-GWO-SVM; 4: VMD-DE-LCPGWO-SVM; 5: VMD-RCMDE-GWO-SVM; 6: VMD-RCMDE-LCPGWO-SVM; 7: VMD-ARCMDE-GWO-SVM; 8: VMD-ARCMDE-LCPGWO-SVM.

In order to show the fault identification evaluation results comparison of different methods more intuitively, the comparison of evaluation values of different methods under 1800 rpm are shown in Figure 8, illustrating that the proposed method has obvious advantage in fault identification. As shown in Figure 9, the proposed VMD-ARCMDE-LCPGWO-SVM method achieved more outstanding results than the other methods on the whole, where the evaluation values of the data had a strong performance at 2200 rpm. Furthermore, the boxplots of the four evaluation values are displayed in Figure 10, it demonstrates the performances of the different methods. The proposed method possesses better stability and overall performance. Therefore, with the experiments on various fault locations, motor speeds and the detailed comparative analysis given above, the superiority of the proposed identification model is effectively demonstrated.

## 5. Conclusions

Increasingly complex rotating machinery equipment must have excellent mechanical fault identification technology to ensure its safe and effective operation. In this paper, a novel fault identification approach is proposed by fusing VMD, ARCMDE and SVM with LCPGWO optimization. Firstly, VMD was employed to decompose the nonstationary fault signals into several IMFs by the center frequency observation method. Afterwards, ARCMDE fusing the superiorities of DE and average refined composite multiscale procedure, was proposed to construct the feature vectors of different fault samples, which performed excellently in multiscale fault feature extraction from the IMFs. Subsequently, LCPGWO, which was GWO enhanced by multistrategy, including levy flight, cosine factor and polynomial mutation strategies, was compared with the other algorithms on different benchmark functions. The results demonstrated that the use of LCPGWO was explored to improve the ability of global search, accelerate convergence and enhance the capacity for jumping out of local optimum in the later iteration. Thus, LCPGWO was applied to optimize penalty factor *C* and kernel parameter *g* of SVM model, which was employed to realize the fault classification for different fault samples. Lastly, the proposed VMD-ARCMDE-LCPGWO-SVM method was applied to compare with other methods for rolling bearing fault identification. Meanwhile, the experiment results were measured by four evaluation metrics named ACC, ARI, F and NMI. The proposed fault identification method has smaller error, better stability and higher reliability than the other contrastive methods. Particularly, under motor speed 1800 rpm, the identification accuracy of the proposed method was 9.33, 3.62, 1.71 and 0.57% higher than the VMD-FE-GWO-SVM, VMD-DE-GWO-SVM, VMD-RCMDE-GWO-SVM and VMD-ARCMDE-GWO-SVM methods; and also 8.09, 3.43 and 0.67 higher than the VMD-FE-LCPGWO-SVM, VMD-DE-LCPGWO-SVM and VMD-RCMDE-LCPGWO-SVM methods. Meanwhile, the evaluation metrics were also outstanding under 2200 rpm. Therefore, it can be expected to provide a new way for rolling bearing fault identification.

## 6. Discussion

The generation and development of rolling bearing faults are caused by the coupling of many factors, which contain a large number of uncertain factors. Conventional diagnostic methods have difficulty in obtaining satisfactory results. The SVM method is a relatively novel method in the field of rolling bearing fault identification. Although some research has been done in this paper, the author believes that there are still some issues worthy of further research: (1) in practical engineering applications, different components in the unit influence each other, complex rolling bearing combinations may have multifactor failures in the future. Therefore, it is still necessary to conduct in depth failure mechanism research to make the identification work more targeted, more accurate and reliable. (2) The research of a fault identification classifier is only one aspect of the problem of fault identification. The premise of fault identification is to apply an advanced signal analysis method to extract more effective and more capable features from the rolling bearings’ operating state. Therefore, it is necessary to extract fault feature information from multiple angles according to the bearing fault signal characteristics and combined with new signal processing technology, so as to lay a foundation for the SVM to provide more effective fault features. (3) The occurrence of rolling bearing faults is a gradual process, and minor failures have little impact, but parts must be replaced after reaching a certain degree of severity. Therefore, real-time monitoring of rolling bearings is very important. This paper only analyzes the bearing vibration signals collected on the experimental platform, and does not realize real-time monitoring, which is also the direction of author’s next research.

## Figures and Tables

**Table 1:** Overview of 12 benchmark functions.

No.	Function	Range	F_min_
1	$F1(x)=\sum_{i=1}^{n}x_{i}^{2}$	[−100, 100]	0
2	$F2(x)=\sum_{i=1}^{n}\left|x_{i}\right|+\prod_{i=1}^{n}\left|x_{i}\right|$	[−10, 10]	0
3	$F3(x)=\sum_{i=1}^{n}(\sum_{j=1}^{i}x_{j}{)}^{2}$	[−100, 100]	0
4	$F4(x)=\max(\left|x_{i}\right|,1\leq i\leq n)$	[−100, 100]	0
5	$F5(x)=\sum_{i=1}^{n-1}\left[100{\left(x_{i+1}-x_{i}^{2}\right)}^{2}+{\left(x_{i}-1\right)}^{2}\right]$	[−30, 30]	0
6	$F6(x)=\sum_{i=1}^{n}ix_{i}^{2}$	[−10, 10]	0
7	$F7(x)=\sum_{i=1}^{n}{\left(10^{6}\right)}^{\left(i-1\right)/\left(n-1\right)}x_{i}^{2}$	[−100, 100]	0
8	$F8(x)=-20e(-0.2\sqrt{\frac{1}{n}\sum_{i=1}^{n}x_{i}^{2}})-e(\frac{1}{n}\sum_{i=1}^{n}\cos(2\pi x_{i}))+20+e$	[−32, 32]	0
9	$F9(x)=\frac{1}{4000}\sum_{i=1}^{n}x_{i}^{2}+\prod_{i=1}^{n}\cos(\frac{x_{i}}{\sqrt{i}})+1$	[−100, 100]	0
10	$F10(x)=\sum_{i=1}^{n}\left(x_{i}^{2}-10\cos(2\pi x_{i})\right)+10n$	[−5.12, 5.12]	0
11	$F11(x)=\sum_{i=1}^{n}\left|x_{i}\sin(x_{i})+0.1x_{i}\right|$	[−10, 10]	0
12	$\begin{array}{c} F12\left(x\right)=\frac{p}{n}\left\{10sin\left(py_{1}\right)+\sum_{i=1}^{n-1}{\left(y_{i}-1\right)}^{2}\left[1+10sin^{2}\left(py_{i+1}\right)\right]+{\left(y_{n}-1\right)}^{2}\right\}\\ +\sum_{i=1}^{n}u\left(x_{i},10,100,4\right)\\ y_{i}=1+\frac{x_{i}+1}{4}u\left(x_{i},a,m\right)=\{\begin{array}{ll} k{\left(x_{i}-a\right)}^{m} & x_{i}>a\\ 0 & -a<x_{i}<a\\ k{\left(-x_{i}-a\right)}^{m} & x_{i}<-a \end{array} \end{array}$	[−50, 50]	0

**Table 2:** Parameter settings of different optimization algorithms.

Models	Parameter	Determination Approach	Range Determined Value
PSO	iteration number	preset	200
	searching agents	preset	40
	dimensions	preset	30
GWO	iteration number	preset	200
	searching agents	preset	40
	dimensions	preset	30
SCA	iteration number	preset	200
	searching agents	preset	40
	dimensions	preset	30
WOA	iteration number	preset	200
	searching agents	preset	40
	dimensions	preset	30
MFO	iteration number	preset	200
	searching agents	preset	40
	dimensions	preset	30
DE	iteration number	preset	200
	searching agents	preset	40
	dimensions	preset	30
LCPGWO	iteration number	preset	200
	searching agents	preset	40
	dimensions	preset	30

**Table 3:** The comparison results of the seven algorithms on benchmark functions.

Function		PSO	GWO	SCA	WOA	MFO	DE	LCPGWO
F1	Max	2.19 × 10^−1^	1.69 × 10^−9^	1.21 × 10^3^	9.99 × 10^−6^	1.86 × 10^3^	6.17 × 10^1^	1.29 × 10^−181^
	Min	2.14 × 10^−2^	6.60 × 10^−11^	4.32 × 10^1^	3.71 × 10^−7^	4.87 × 10^2^	2.28 × 10^1^	6.51 × 10^−183^
	Mean	1.17 × 10^−1^	3.62 × 10^−10^	4.33 × 10^2^	3.12 × 10^−6^	1.14 × 10^3^	3.46 × 10^1^	3.04 × 10^−182^
	Std	6.24 × 10^−2^	4.87 × 10^−10^	4.43 × 10^2^	3.07 × 10^−6^	4.22 × 10^2^	1.18 × 10^1^	0.00
F2	Max	2.22 × 10^0^	1.34 × 10^−6^	3.79 × 10^0^	1.28 × 10^−4^	5.33 × 10^1^	2.22 × 10^0^	1.82 × 10^−83^
	Min	4.04 × 10^−1^	7.07 × 10^−7^	2.46 × 10^−1^	2.19 × 10^−5^	1.03 × 101	1.52 × 10^0^	1.20 × 10^−84^
	Mean	9.68 × 10^−1^	9.95 × 10^−7^	1.42 × 10^0^	4.85 × 10^−5^	2.99 × 10^1^	1.75 × 10^0^	7.58 × 10^−84^
	Std	5.24 × 10^−1^	1.88 × 10^−7^	1.07 × 10^0^	3.25 × 10^−5^	1.39 × 10^1^	2.10 × 10^−1^	5.04 × 10^−84^
F3	Max	5.36 × 10^2^	5.26 × 10^0^	3.16 × 10^4^	1.03 × 10^2^	4.52 × 10^4^	4.83 × 10^4^	1.01 × 10^−180^
	Min	2.45 × 10^2^	3.78 × 10^−2^	4.76 × 10^3^	2.36 × 10^0^	1.47 × 10^4^	3.31 × 10^4^	1.34 × 10^−182^
	Mean	3.48 × 10^2^	1.63 × 10^0^	1.70 × 10^4^	3.51 × 10^1^	2.50 × 10^4^	4.28 × 10^4^	2.42 × 10^−181^
	Std	1.02 × 10^2^	1.68 × 10^0^	7.81 × 10^3^	3.39 × 10^1^	9.84 × 10^3^	4.83 × 10^3^	0.00
F4	Max	2.75 × 10^0^	5.87 × 10^−2^	6.53 × 10^1^	8.65 × 10^−1^	8.06 × 10^1^	4.08 × 10^1^	8.75 × 10^−97^
	Min	1.88 × 10^0^	5.03 × 10^−3^	2.88 × 10^1^	1.11 × 10^−1^	5.16 × 10^1^	3.49 × 10^1^	2.56 × 10^−97^
	Mean	2.10 × 10^0^	1.80 × 10^−2^	5.27 × 10^1^	3.37 × 10^−1^	6.50 × 10^1^	3.79 × 10^1^	5.05 × 10^−97^
	Std	2.58 × 10^−1^	1.77 × 10^−2^	1.32 × 10^1^	2.68 × 10^−1^	9.07 × 10^0^	1.98 × 10^0^	2.11 × 10^−97^
F5	Max	5.11 × 10^2^	2.88 × 10^1^	1.38 × 10^7^	2.86 × 10^1^	1.20 × 10^6^	1.05 × 10^4^	2.24 × 10^1^
	Min	6.75 × 10^1^	2.62 × 10^1^	5.75 × 10^4^	2.61 × 10^1^	1.02 × 10^5^	3.57 × 10^3^	1.00 × 10^1^
	Mean	1.86 × 10^2^	2.76 × 10^1^	2.86 × 10^6^	2.75 × 10^1^	5.73 × 10^5^	6.52 × 10^3^	1.67 × 10^1^
	Std	1.33 × 10^2^	9.23 × 10^−1^	4.50 × 10^6^	8.08 × 10^−1^	3.68 × 10^5^	2.24 × 10^3^	4.11 × 10^0^
F6	Max	7.95 × 10^0^	2.33 × 10^−10^	1.47 × 10^2^	7.43 × 10^−6^	1.95 × 10^3^	6.83 × 10^0^	1.30 × 10^−188^
	Min	5.82 × 10^−1^	9.51 × 10^−12^	3.23 × 10^0^	1.64 × 10^−8^	8.74 × 10^1^	3.47 × 10^0^	1.22 × 10^−189^
	Mean	1.77 × 10^0^	7.16 × 10^−11^	5.54 × 10^1^	1.17 × 10^−6^	6.96 × 10^2^	4.72 × 10^0^	6.08 × 10^−189^
	Std	2.19 × 10^0^	6.86 × 10^−11^	4.91 × 10^1^	2.26 × 10^−6^	5.81 × 10^2^	9.98 × 10^−1^	0.00
F7	Max	4.26 × 10^4^	8.55 × 10^−7^	3.90 × 10^5^	1.15 × 10^−2^	1.54 × 10^8^	8.14 × 10^4^	3.81 × 10^−166^
	Min	1.16 × 10^3^	1.80 × 10^−7^	4.57 × 10^3^	6.78 × 10^−4^	1.42 × 10^6^	3.80 × 10^4^4	1.25 × 10^−167^
	Mean	7.97 × 10^3^	5.56 × 10^−7^	1.09 × 10^5^	4.09 × 10^−3^	2.93 × 10^7^	6.39 × 10^4^	1.49 × 10^−166^
	Std	1.26 × 10^4^	2.28 × 10^−7^	1.11 × 10^5^	3.72 × 10^−3^	4.52 × 10^7^	1.56 × 10^4^	0.00
F8	Max	1.66 × 10^0^	4.62 × 10^−6^	2.04 × 10^1^	2.04 × 10^1^	1.99 × 10^1^	3.75 × 10^0^	7.99 × 10^−15^
	Min	1.75 × 10^−1^	1.57 × 10^−6^	3.45 × 10^0^	4.58 × 10^−5^	7.53 × 10^0^	2.95 × 10^0^	4.44 × 10^−15^
	Mean	1.12 × 10^0^	3.36 × 10^−6^	1.34 × 10^1^	6.07 × 10^0^	1.50 × 10^1^	3.38 × 10^0^	6.57 × 10^−15^
	Std	4.63 × 10^−1^	1.03 × 10^−6^	7.40 × 10^0^	9.78 × 10^0^	5.24 × 10^0^	2.46 × 10^−1^	1.83 × 10^−15^
F9	Max	4.96 × 10^−2^	7.78 × 10^−2^	2.06 × 10^0^	2.77 × 10^−2^	1.55 × 10^0^	9.73 × 10^−1^	0.00
	Min	5.88 × 10^−3^	2.21 × 10^−12^	8.26 × 10^−1^	3.56 × 10^−8^	1.11 × 10^0^	7.73 × 10^−1^	0.00
	Mean	1.94 × 10^−2^	1.08 × 10^−2^	1.13 × 10^0^	9.18 × 10^−3^	1.29 × 10^0^	8.71 × 10^−1^	0.00
	Std	1.22 × 10^−2^	2.44 × 10^−2^	3.65 × 10^−1^	1.06 × 10^−2^	1.21 × 10^−1^	6.18 × 10^−2^	0.00
F10	Max	1.63 × 10^2^	3.02 × 10^1^	1.61 × 10^2^	4.41 × 10^1^	2.55 × 10^2^	1.44 × 10^2^	0.00
	Min	6.25 × 10^1^	6.38 × 10^0^	2.92 × 10^1^	8.63 × 10^0^	1.17 × 10^2^	1.22 × 10^2^	0.00
	Mean	9.41 × 10^1^	1.60 × 10^1^	6.56 × 10^1^	2.09 × 10^1^	1.76 × 10^2^	1.32 × 10^2^	0.00
	Std	3.17 × 10^1^	7.64 × 10^0^	3.79 × 10^1^	1.14 × 10^1^	4.04 × 10^1^	7.99 × 10^0^	0.00
F11	Max	2.76 × 10^0^	5.67 × 10^−3^	1.18 × 10^1^	2.36 × 10^0^	1.63 × 10^1^	9.46 × 10^0^	2.54 × 10^−2^
	Min	6.25 × 10^−1^	2.34 × 10^−3^	3.03 × 10^−1^	2.40 × 10^−3^	4.10 × 10^0^	6.46 × 10^0^	7.47 × 10^−69^
	Mean	1.55 × 10^0^	3.58 × 10^−3^	4.85 × 10^0^	5.15 × 10^−1^	9.40 × 10^0^	7.99 × 10^0^	2.54 × 10^−3^
	Std	7.38 × 10^−1^	1.11 × 10^−3^	4.44 × 10^0^	7.79 × 10^−1^	4.08 × 10^0^	1.09 × 10^0^	8.03 × 10^−3^
F12	Max	−4.61 × 10^2^	−5.78 × 10^2^	−4.86 × 10^2^	−7.67 × 10^2^	−9.95 × 10^2^	−1.04 × 10^3^	−1.06 × 10^3^
	Min	−9.78 × 10^2^	−7.16 × 10^2^	−5.88 × 10^2^	−8.71 × 10^2^	−1.06 × 10^3^	−1.06 × 10^3^	−1.06 × 10^3^
	Mean	−6.96 × 10^2^	−6.39 × 10^2^	−5.29 × 10^2^	−8.24 × 10^2^	−1.05 × 10^3^	−1.06 × 10^3^	−1.06 × 10^3^
	Std	1.39 × 10^2^	4.53 × 10^1^	3.40 × 10^1^	3.36 × 10^1^	2.14 × 10^1^	7.94 × 10^0^	2.40 × 10^−13^

**Table 4:** Description of the experimental data.

Motor Speed	Fault Position	Number of Total Samples	Number of Training Samples	Number of Testing Samples	Label
1800 rpm	Normal	61	40	21	L1
	Inner race	61	40	21	L2
	Outer race	61	40	21	L3
	Ball fault	61	40	21	L4
	Combination fault	61	40	21	L5
2200 rpm	Normal	61	40	21	L6
	Inner race	61	40	21	L7
	Outer race	61	40	21	L8
	Ball fault	61	40	21	L9
	Combination fault	61	40	21	L10

**Table 5:** The parameter settings of ARCMDE.

**Parameter**	$\tau_{\max}$	*m*	*c*	$t_{d}$
**Value**	20	4	6	1

**Table 6:** The calculation method of the four metrics.

Abbreviation	Expression
ACC	$ACC=\frac{TP+TN}{TP+FN+FP+FN}$
ARI	$ARI=\frac{n_{11}+n_{00}}{C_{n}^{2}}$
F	$F=2\frac{\left(TP/\left(TP+FP\right)\right)\left(TP/\left(TP+FN\right)\right)}{TP/\left(TP+FP\right)+TP/\left(TP+FN\right)}$
NMI	$NMI=\frac{\sum_{\varphi \in \Phi }\sum_{\omega \in \Omega }P\left(\varphi ,\omega \right)log\left(P\left(\varphi ,\omega \right)/P\left(\varphi \right)P\left(\omega \right)\right)}{\sqrt{\left(\sum_{\varphi \in \Phi }P\left(\varphi \right)log\left(P\left(\varphi \right)\right)\right)\left(\sum_{\omega \in \Omega }P\left(\omega \right)log\left(P\left(\omega \right)\right)\right)}}$

**Table 7:** Comparison results with different methods under variable sampling speeds.

Motor Speed	Methods	Best C	Best g	Evaluation Metrics			
				ARI	NMI	F	ACC
1800 rpm	VMD-FE-GWO-SVM	334.3608	13.2605	0.7697 [−0.0439, 0.0532]	0.8142 [−0.0477, 0.0545]	0.8885 [−0.0286, 0.0264]	0.8905 [−0.0238, 0.0238]
	VMD-FE-LCPGWO-SVM	14.792	5.8259	0.7919 [−0.1008, 0.0650]	0.8360 [−0.0202, 0.0620]	0.9004 [−0.06130, 0.0320]	0.9029 [−0.0553, 0.0304]
	VMD-DE-GWO-SVM	2.3934	21.5967	0.8748 [−0.0544, 0.0775]	0.8845 [−0.0568, 0.0678]	0.9473 [−0.0240, 0.0334]	0.9476 [−0.0238, 0.0340]
	VMD-DE-LCPGWO-SVM	166.1041	38.8857	0.8791 [−0.0868, 0.0508]	0.8906 [−0.0732, 0.0382]	0.9490 [−0.0335, 0.0222]	0.9495 [−0.0352, 0.0219]
	VMD-RCMDE-GWO-SVM	445.5278	0.0353	0.9202 [−0.0716, 0.0327]	0.9298 [−0.0563, 0.0305]	0.9658 [−0.0371, 0.0151]	0.9667 [−0.0334, 0.0143]
	VMD-RCMDE-LCPGWO-SVM	708.8285	0.2694	0.9439 [−0.0348, 0.0319]	0.9488 [−0.0246, 0.0273]	0.9769 [−0.0159, 0.0136]	0.9771 [−0.0152, 0.0134]
	VMD-ARCMDE-GWO-SVM	683.77	0.25	0.9458 [−0.0639, 0.0300]	0.9500 [−0.0533, 0.0261]	0.9780 [−0.0248, 0.0125]	0.9781 [−0.0257, 0.0124]
	VMD-ARCMDE-LCPGWO-SVM	5.6124	0.2451	0.9597 [−0.0310, 0.0403]	0.9627 [−0.0342, 0.0373]	0.9838 [−0.0124, 0.0162]	0.9838 [−0.0124, 0.0162]
2200 rpm	VMD-FE-GWO-SVM	43.6013	0.4489	0.7216 [−0.1137,0.0993]	0.7573 [−0.0814,0.0705]	0.8725 [−0.0512,0.0509]	0.8733 [−0.0543,0.0505]
	VMD-FE-LCPGWO-SVM	72.1392	0.8806	0.7327 [−0.0991,0.0919]	0.7640 [−0.0868,0.0831]	0.8761 [−0.0472,0.0458]	0.8781 [−0.0495,0.0457]
	VMD-DE-GWO-SVM	20.573	27.4512	0.8604 [−0.0651, 0.0447]	0.8732 [−0.0410, 0.0392]	0.9417 [−0.0263, 0.0202]	0.9419 [−0.0276, 0.0200]
	VMD-DE-LCPGWO-SVM	5.5	69.6902	0.8652 [−0.0663, 0.0877]	0.8815 [−0.0544, 0.0788]	0.9439 [−0.0316, 0.0370]	0.9438 [−0.0295, 0.0371]
	VMD-RCMDE-GWO-SVM	653.1094	0.4638	0.9220 [−0.0535, 0.0538]	0.9330 [−0.0346, 0.0430]	0.9675 [−0.0249, 0.0230]	0.9676 [−0.0248, 0.0229]
	VMD-RCMDE-LCPGWO-SVM	408.9463	0.2012	0.9242 [−0.0557, 0.0287]	0.9337 [−0.0352, 0.0267]	0.9684 [−0.0258, 0.0125]	0.9686 [−0.0257, 0.0124]
	VMD-ARCMDE-GWO-SVM	767.9240	0.0013	0.9271 [−0.0401, 0.0487]	0.9385 [−0.0377, 0.0376]	0.9693 [−0.0176, 0.0212]	0.9695 [−0.0171, 0.0210]
	VMD-ARCMDE-LCPGWO-SVM	172.4596	0.0185	0.9303 [−0.0461, 0.0455]	0.9381 [−0.0380, 0.0380]	0.9712 [−0.0207, 0.0193]	0.9714 [−0.0190, 0.0191]

## Data Availability

The data included in this study are all owned by the research group and will not be transmitted.
